# Opioid rotation versus combination for cancer patients with chronic uncontrolled pain: a randomized study

**DOI:** 10.1186/s12904-015-0038-7

**Published:** 2015-09-16

**Authors:** Hyun-Jun Kim, Young Saing Kim, Se Hoon Park

**Affiliations:** Department of Obstetrics and Gynecology, Konkuk University, Chungju, South Korea; Department of Internal Medicine, Gachon University Gil Medical Center, Incheon, South Korea; Department of Medicine, Sungkyunkwan University Samsung Medical Center, 135-710 Seoul, South Korea

**Keywords:** Cancer, Pain, Brief pain inventory, Opioid rotation, Opioid combination

## Abstract

**Background:**

For cancer patients with inadequate pain relief, a switch to an alternative opioid is the preferred option for symptomatic improvement. However, multiple opioids are often simultaneously administered for anecdotal reasons. This prospective study evaluated pain response to either opioid rotation or combination in patients with uncontrolled cancer pain.

**Methods:**

Patients suffering with uncontrolled cancer pain despite dose titration were randomly assigned to opioid rotation group or opioid combination group. Patients answered a questionnaire that included items on pain severity (0 to 10) and interferences at baseline and after one week.

**Results:**

Of the 50 patients registered, 39 patients answered the questionnaire after one week of treatment. After one week, the mean pain scores were significantly improved in both groups. Ten patients (42 %) in the rotation group and 16 patients (62 %) in the combination group reported that they achieved relief from pain (*p* = 0.08). The incidence of adverse events was similar in both groups, but fewer patients experienced constipation with opioid rotation than with combination (17 % *vs*. 42 %, respectively; *p* = 0.05). The frequency of rescue analgesics (50 % *vs*. 69 %; *p* = 0.17) and dose modification (29 % *vs*. 38 %; *p* = 0.49) were similar in the rotation and combination groups.

**Conclusions:**

For patients with chronic uncontrolled cancer pain, both opioid rotation and combination strategies appear to provide significant relief of pain and improved patient satisfaction.

**Trial registration:**

This study was registered in advance to ClinicalTrials.gov (no. NCT00478101).

## Background

Chronic opioid therapy is the mainstay treatment for moderate to severe cancer pain. Experts agree that patients suffering with cancer-related pain should be treated with strong opioids as soon as the non-opioid analgesics become ineffective [1]. However, many cancer patients continue to suffer either because their pain is undertreated, or because of the adverse effects of opioids [2, 3]. If pain relief and improved functionality are not demonstrated, then other types of medication should be considered along with alternative analgesics to achieve patient-specific pain goals. Thus, opioid rotation is becoming an established treatment for these patients with uncontrolled pain [4]. The underlying rationales behind opioid rotation are incomplete cross tolerance and the possibility of improved pain control through achieving maximum dose titration of the opioid in the patients with intrinsically opioid-responsive pain [5]. However, it is often impossible to know in advance the efficacy of the chosen alternative opioid chosen because this depends on a series of factors, including the individual response, the pain mechanism, and the degree of cross tolerance [4].

Anecdotal reports have shown that multiple opioids are often simultaneously administered for different reasons [6], other than the obvious disparity in effecting different receptor subgroups and specificity. In a randomized study [7], the rescue morphine consumption was much higher in patients who received morphine only, compared to the patients who received morphine and oxycodone, suggesting that the opioid combination can be a useful alternative for uncontrolled, severe cancer pain, resulting in a better analgesia profile and less toxicity.

Although morphine is usually considered the preferred drug for the treatment of severe cancer pain, the use of transdermal fentanyl has been increasing in recent years. Transdermal fentanyl is a synthetic opioid originally that was used as a component of anesthetic regimens, and it provides patients with continuous systemic delivery of analgesic effect at a controlled rate for up to 72 h: this makes it suited for the treatment of chronic pain [8]. More recently, some studies have demonstrated that transdermal fentanyl can be effectively and safely administered to patients with cancer pain, regardless of whether they have previously received opioids [9–11]. Based these data, the present study was designed to assess the analgesic profiles of two different strategies for controlling chronic cancer pain: the opioid rotation to transdermal fentanyl, and the combination of oral oxycodone and transdermal fentanyl.

## Methods

We performed a prospective study on a sample of consecutive patients who were admitted to a palliative cancer care unit for a period of 10 months. Patients were eligible to participate in the study if their clinician considered they had chronic uncontrolled pain associated with a histologically confirmed solid cancer that required stronger opioid therapy than they had been taking and they were over 18 years of age. Patients receiving oral opioids only were eligible. Chronic uncontrolled pain was defined if a patient had persistent pain despite adequate dose titration and being treated with oral morphine equivalent of 100 mg/d or more. Daily opioid requirement was calculated as the total amount of opioids administered including both long- and short-acting opioids. Patients were excluded if they were suspected to have narcotic abuse, clinically relevant CO_2_ retention, an active skin disease that precluded the use of transdermal patches, or on antitumor therapy of any kind. Other exclusion criteria included the inability to swallow oral medication, and impaired sensory or cognitive function. Patients who had an active infection, or uncontrolled central nervous system involvement were also excluded. All patients gave their written informed consent before participating in the study and the protocol was reviewed and approved by the Gil Medical Center (Incheon, Korea) institutional review board.

Following a screening procedure, the patients who completed the baseline questionnaire were randomly assigned to the rotation group (to transdermal fentanyl) or the combination group (oral oxycodone plus transdermal fentanyl). Patients who did not require a change in analgesic dosing during the screening period were considered ineligible. The dosing of transdermal fentanyl was mainly based on the National Comprehensive Cancer Network (NCCN) practice guidelines (adult cancer pain v2.2005), and on the department policy. These guidelines required a working knowledge of an equianalgesic dose table [12]. An individualized equianalgesic dosing algorithm was calculated for each patient on the basis of the history of opioid administration before registration. According to the algorithms, the following conversion ratios were used: oral morphine 100 mg = oral oxycodone 66 mg = q72h dose of 50 mcg/h transdermal fentanyl. Because the patients were suffering uncontrolled severe pain, the first decision was made to increase the starting dose by 25 %. The patients allocated to the combination group were switched into transdermal fentanyl every 3 days (half of the desired daily opioid dose) plus oral oxycodone every 8 to 12 h. For practical reason, the transdermal fentanyl doses were rounded down to the nearest dose that could be administered with 12.5 mcg/h, 25 mcg/h and 50 mcg/h patches of the drug. Breakthrough pain, if any, was controlled with immediate-release oxycodone. Concomitant administration of non-opioid analgesics or corticosteroids was permitted provided that the doses were constant before and throughout the study.

Patients answered a questionnaire that included items concerned with pain severity and interference with the activities of daily living from the modified short form of the Brief Pain Inventory [13]. Pain was rated with a numerical rating scale (NRS) that ranged from 0 (no pain at all) to 10 (the worst pain you can imagine) [14]. We evaluated the changes in pain and interference items between the baseline and after the 7-days of therapy. The primary outcomes were changes in the pain score and treatment success. Treatment success was achieved when the pain NRS decreased by at least 33 % of the baseline value recorded before randomization. Treatment failure was followed by alternative measures, including a further addition of different opioids, according to the decision by clinicians. If the patient completed only the baseline questionnaire, treatment outcome was classified as a failure. Patients’ satisfaction, the number of rescue opioid doses, the overall well-being using a 0 to 10 NRS and adverse events were the secondary measures of efficacy and tolerability. Adverse events were measured at baseline and then daily until the treatment success or failure was documented.

This randomized study was treated, statistically, as two simultaneous phase II studies and the one-stage design was applied separately for each treatment group. At a two or more change of the pain NRS from baseline and with a standard deviation of 2 for the two groups, the required number of patients was calculated to be 21 per group (two-sided significance of 0.05 and a power of 90 %). For the primary outcome in each group, differences in the mean changes of the pain NRS between the groups were assessed by using a *t*-test. Although no direct between-group comparison of outcomes was planned, we investigated, for exploratory purposes, the effects of opioid rotation and combination by means of *t*-test and Fisher’s exact test. The results are presented as means ± SDs. All analyses were performed on the per-protocol population, which was defined as all patients who answered the baseline questionnaire and who took at least one dose of the study drug.

## Results

A total of 50 patients were randomized to the rotation group (*n* = 24) or the combination group (*n* = 26). A summary of the demographic and baseline disease characteristics is presented in Table 1. There were no significant differences between groups with respect to the baseline characteristics. Patients had a median age of 66 years and they were predominantly male (70 %). The mean baseline pain scores were similar for the rotation and combination groups (Table 2). All patients had been treated with oral opioids and their baseline analgesic doses were similar for the two groups (median, 120 and 128 mg of oral morphine equivalent, respectively). Of 50 patients who completed baseline questionnaire, 39 patients (rotation group, *n* = 20 vs. combination group, *n* = 19) answered the questionnaire after one week of treatment.

**Table 1:** Baseline characteristics

	Rotation group	Combination group
No. of patients	24	26
Age, years		
Median (range)	67 (27–72)	62 (37–83)
Male gender	16 (67 %)	19 (73 %)
Primary diagnosis		
Gastrointestine	9	4
Head and neck	8	5
Lung	6	5
Sarcoma	0	5
Hepatobiliary	0	4
Brain	0	3
Breast	1	0
Baseline analgesics, median (range)		
Oral morphine equivalent (mg/day)	120 (80–240)	128 (76–208)

**Table 2:** Changes in the pain-related parameters

	Rotation group		Combination group	
	Baseline (*n* = 24)	Day 7 (*n* = 20)	Baseline (*n* = 26)	Day 7 (*n* = 19)
Pain (mean ± SD)				
Maximal pain	6.2 ± 2.2	4.7 ± 2.4	6.5 ± 1.8	4.8 ± 2.1
Minimal pain	2.9 ± 2.8	2.0 ± 1.9	3.0 ± 2.5	1.8 ± 1.5
Average pain	4.5 ± 2.8	3.4 ± 2.8	4.6 ± 1.8	2.9 ± 1.6
Current pain	5.3 ± 3.1	3.4 ± 2.9	4.7 ± 2.2	2.5 ± 1.7
Relief from pain	2 (8 %)	10 (42 %)	6 (23 %)	16 (62 %)
Interference (mean ± SD)				
General activity	6.9 ± 3.3	6.2 ± 2.8	5.9 ± 3.6	6.5 ± 2.2
Mood	6.0 ± 3.8	5.3 ± 3.4	7.1 ± 3.1	5.5 ± 3.0
Walking ability	4.3 ± 4.2	3.8 ± 3.9	5.7 ± 3.3	4.7 ± 3.2
Normal work	5.0 ± 4.6	4.5 ± 3.9	6.4 ± 3.3	6.2 ± 3.8
Relation with others	5.2 ± 4.4	6.4 ± 3.6	6.7 ± 3.7	6.7 ± 3.6
Sleep	4.0 ± 4.1	4.1 ± 3.9	4.7 ± 3.6	4.7 ± 3.7
Enjoyment of life	6.7 ± 3.9	7.8 ± 3.1	7.8 ± 2.9	8.3 ± 2.1

**Fig. 1:**
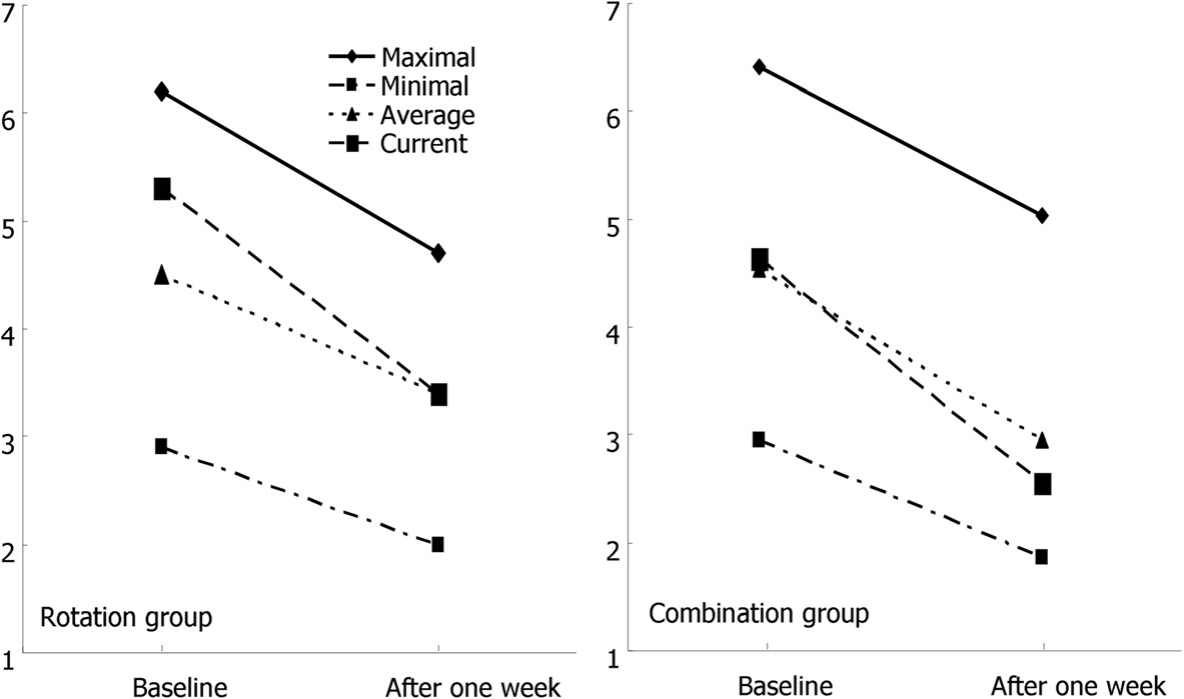
Changes in the pain scores

As shown in Table 2 and Fig. 1, the pain scores after 7-days of treatment were significantly improved in both groups. The maximal pain scores in the rotation group decreased from 6.2 (±2.2) at baseline to 4.7 (±2.4) on day 8 (*p* = 0.03), compared with decrease from 6.5 (±1.8) to 4.8 (±2.1) in the combination group (*p* < 0.01). The current pain scores were significantly decreased in the rotation group (5.3 to 3.4; *p* = 0.04) and also in the combination group (4.7 to 2.5; *p* < 0.01). The mean NRS for minimal pain was decreased from 2.9 to 2.0 in the rotation group (*p* = 0.22) and from 3.0 to 1.8 in the combination group (*p* = 0.06). The average pain scores were also decreased in the rotation group (4.5 to 3.4; *p* = 0.18) and in the combination group (4.6 to 2.9; *p* < 0.01). Treatment success was achieved by 11 rotation group patients and 12 combination group patients (*p* = 0.98).

For the secondary efficacy outcomes, more patients in the combination group answered that they had achieved relief from pain after 7 days of treatment (42 for the rotation group and 62 % for the combination group; *p* = 0.09), although this was statistically insignificant. Rescue analgesics were required in 50 and 69 % of patients in the rotation group and combination group, respectively (*p* = 0.17). During 7-days of the treatment period, modification in the analgesic dosing was done for 29 and 38 % of the patients in the rotation group and combination group, respectively (*p* = 0.49). The mean baseline NRS for each category of interference items (general activity, mood, walking, work, social relation, sleep and enjoyment of life) were similar for both groups (Table 2).

The median fentanyl doses were 50 mcg/h (range, 25 to 200 mcg/h) in the rotation group and 25 mcg/h (range, 25 to 100 mcg/h) in the combination group. In the combination group, the median oxycodone dose was 30 mg/d (range, 20 to 80 mg/d). One patient in the rotation group died of massive upper gastrointestinal bleeding shortly after starting the study drug. This event was thought unlikely to be related to the study drug because his primary tumor was remained in the stomach. Treatment-related adverse events, which were defined as those events deemed to be related to the study drugs, were reported in 50 % of the rotation group and 54 % of the combination group patients. The incidence of adverse events was similar; however, more patients experienced constipation with opioid combination (42 %) than with rotation (17 %; *p* = 0.05). Of the original 50 patients, 5 (21 %) of the rotation group patients and 6 (23 %) of the combination group patients discontinued treatment before completing the study. Clinical deterioration of their general condition was the most common reason for discontinuation (*n* = 6). A similar number of patients withdrew treatment owing to adverse events (*n* = 2) or inadequate pain control (*n* = 3).

## Discussion

This randomized, but not double-blind, phase II study was aimed at testing two different methods of treatment. A double-blind study is particularly difficult to realize when different delivery systems (i.e., oral and transdermal) are used. For the patients with chronic uncontrolled cancer pain, both opioid rotation and combination strategies appear to provide significant relief of pain and patient satisfaction. Although our trial was not intended to detect differences between the two groups, the efficacy of opioid rotation and opioid combination did not seem to differ in terms of pain control and the patient satisfaction. The clinical significance of these findings was supported by the secondary efficacy outcomes that measured relief of pain and the interference with the activities of daily living.

The medical literature does not recommend the simultaneous use of two strong opioids for chronic cancer pain [15, 16]. It is regarded as seldom advantageous to combine two different opioids to treat pain. Currently, the rationale for prescribing more than one strong opioids (e.g., oral oxycodone and transdermal fentanyl) is limited and appears to be a duplication of therapy. There are few clear, clinically supported rationales in the published literature to support the use of the two strong opioids together. However, such combination therapy of two (or more) strong opioids is a common and real clinical situation, probably because of clinicians’ reluctance to increase the dose of a single opioid to an effective one. Patients may be at increased risk for adverse events, particularly if far-advanced disease or comorbid conditions such as heart failure, obesity, severe asthma, or respiratory difficulty exist. Patients with end-stage cancer that was heavily treated may also be at a risk.

The combination of transdermal fentanyl and oral oxycodone was well tolerated by cancer patients. After one week of treatment, the pain scores were significantly improved in both groups. More patients in the combination group (62 %), although statistically insignificant, reported that they achieved relief from pain than those in the rotation group (42 %). This observation should be interpreted with caution because it represents only a small group of patients with uncontrolled cancer pain and no formal comparisons of outcomes were planned between the two groups. The present study was not designed to compare the synergistic analgesic effect of two strong opioids to that of a single one. However, our results indicate that the combination of transdermal fentanyl and oral oxycodone could be a reasonable option for treating patients with chronic uncontrolled cancer pain. Fentanyl binds only the mu opioid receptor, while oxycodone is a putative kappa receptor agonist. It is possible that the synergistic analgesic effect may be the result of the simultaneous activation of both the mu and kappa opioid receptors. It was reported that the co-administration of sub-analgesic doses of oxycodone and morphine resulted in excellent pain relief with a reduction in opioid-related adverse events [17]. In addition, other reports have indicated that simultaneous administration of one opioid via two routes can result in analgesic synergy in animals [18, 19].

Although there is little controversy regarding the therapeutic efficacy of opioids for controlling chronic cancer pain, patient monitoring and continual reassessment are the keys to providing adequate relief of pain. The beneficial analgesic effect of opioids and the risk associated with their use can vary considerably among individuals. Some patients may not achieve adequate pain relief despite appropriate dose adjustments, whereas others may develop intolerable adverse events to a particular opioid. Therefore, opioid therapy must be individualized such that each dose would provide a balance between effective pain control and acceptable adverse events. To achieve this goal, both opioid rotation and combination therapy could be tried for all patients. The potential benefits and risks should be discussed with the patients.

## Conclusions

In summary, the present study demonstrated that both opioid rotation and combination strategies showed discrete therapeutic efficacy for cancer patients suffering with chronic uncontrolled pain. Taking into account all the uncertainties described above, more studies are needed to investigate whether there are real differences between opioid rotation and combination with respect to the efficacy and toxicity and their combination with other agents. Since no opioid combination regimen has yet reached the level of an evidence-based standard treatment, further research efforts, including studies on combination with other opioids and/or non-opioid analgesics, are encouraged as there continues to be an urgent need to improve our therapeutic strategies against cancer pain.

## Abbreviation

NRS: Numerical rating scale
